# Effects of Different Types of Intermittent Fasting Interventions on Metabolic Health in Healthy Individuals (EDIF): A Randomised Trial with a Controlled-Run in Phase

**DOI:** 10.3390/nu16081114

**Published:** 2024-04-10

**Authors:** Daniel Herz, Sebastian Karl, Johannes Weiß, Paul Zimmermann, Sandra Haupt, Rebecca Tanja Zimmer, Janis Schierbauer, Nadine Bianca Wachsmuth, Maximilian Paul Erlmann, Tobias Niedrist, Kayvan Khoramipour, Thomas Voit, Sian Rilstone, Harald Sourij, Othmar Moser

**Affiliations:** 1 Division of Exercise Physiology and Metabolism, BaySpo—Bayreuth Center of Sport Science, University of Bayreuth, 95440 Bayreuth, Germany; daniel.herz@uni-bayreuth.de (D.H.); sebastian.karl@uni-bayreuth.de (S.K.); johannes.w.weiss@uni-bayreuth.de (J.W.); paul.zimmermann@uni-bayreuth.de (P.Z.); sandra.haupt@uni-bayreuth.de (S.H.); rebecca.zimmer@uni-bayreuth.de (R.T.Z.); janis.schierbauer@uni-bayreuth.de (J.S.); nadine.wachsmuth@uni-bayreuth.de (N.B.W.); maximilian.erlmann@uni-bayreuth.de (M.P.E.); thomas.voit@uni-bayreuth.de (T.V.); sian.rilstone@uni-bayreuth.de (S.R.); 2 Department of Cardiology, Klinikum Bamberg, 96049 Bamberg, Germany; 3 Interdisciplinary Center of Sportsmedicine Bamberg, Klinikum Bamberg, 96049 Bamberg, Germany; 4 Faculty of Life Science—Food, Nutrition & Health, Chair of Molecular Exercise Physiology, University of Bayreuth, 95326 Kulmbach, Germany; 5 Clinical Institute for Medical and Chemical Laboratory Diagnostics, Medical University of Graz, 8036 Graz, Austria; tobias.niedrist@medunigraz.at; 6 Neuroscience Research Center, Institute of Neuropharmacology, Kerman University of Medical Sciences, Kerman 76198-13159, Iran; k.khoramipour@gmail.com; 7 Department of Metabolism, Digestion and Reproduction, Imperial College London, London SW7 2AZ, UK; 8 Interdisciplinary Metabolic Medicine Research Group, Division of Endocrinology and Diabetology, Department of Internal Medicine, Medical University of Graz, 8036 Graz, Austria; ha.sourij@medunigraz.at

**Keywords:** time-restricted feeding, anthropometry, weight loss, metabolism, body mass index

## Abstract

The effects of intermittent fasting (IF) on health promotion in the healthy population remain controversial. Therefore, our study aimed to analyse the efficacy and feasibility of different IF protocols and evaluated the effects within a cohort with a controlled-run in phase on the body mass index (BMI) as the primary outcome, the body composition, and metabolic and haematological markers in healthy participants. A total of 25 individuals were randomised into three fasting groups: 16/8 fasting (*n* = 11), 20/4 fasting (*n* = 6), and alternate-day fasting (ADF, *n* = 8). Assessments were conducted at baseline (visit 1), after a four-week controlled-run in phase (visit 2), and after eight weeks of fasting (visit 3). Both the BMI (*p* = 0.01) and bodyweight (*p* = 0.01) were significantly reduced in the ADF group, which was not seen in the 16/8 and 20/4 groups (*p* > 0.05). Adherence was different but not statistically among the groups (16/8: 84.5 ± 23.0%; 20/4: 92.7 ± 9.5%; and ADF: 78.1 ± 33.5%, *p* = 0.57). Based on our obtained results, the data suggest that some fasting interventions might be promising for metabolic health. However, adherence to the specific fasting protocols remains challenging even for the healthy population.

## 1. Introduction

The global prevalence of overweight and obesity is constantly increasing, as indicated by the World Obesity Federation’s Atlas 2023, predicting that 51% of the world’s population, i.e., more than 4 billion people, will be overweight or obese in the next 12 years [1]. Research has assessed weight loss strategies in recent decades [2]. Among these, a prudent approach called “fasting” has emerged as a potential solution to combat the obesity pandemic [2]. Intermittent fasting (IF) has established itself as one of the most promising fasting methods, which refers basically to refraining from eating for a certain period of time. The most common forms of IF are time-restricted feeding (TRF), such as fasting for 16 h and consuming calories for the remaining 8 hours (16/8). An extended method of IF 16/8 is the 20/4 method, wherein individuals fast for 20 h with a 4 h window for caloric intake. Another typical form of IF is alternate-day fasting (ADF), which involves fasting for 24 h every second day, while caloric intake is allowed on the other days [3].

Based on a systematic review, 27 trials of IF demonstrated weight loss ranging from 0.8% to 13.0% of the initial weight, with no serious adverse events reported. Comparing IF to caloric restriction across twelve studies showed similar effectiveness despite protocol variations [4]. However, a direct parallel group comparison is missing. Further positive effects of IF were shown, leading to a weight loss ranging from 2.5% to 9.9% in obese men over fasting periods between 4 and 30 weeks. Participants in the IF group underwent 16 weeks of energy restriction via eight blocks of 2-week energy restriction interspersed with seven blocks of 2-week energy equalisation [5,6]. It is also important to determine whether the positive effects of IF on body anthropometry can be transferred to healthy individuals. In a study conducted by Heilbronn et al. [6], normal-weight men and women (body mass index (BMI): 20.0–30.0 kg/m^2^) followed an ADF protocol over a period of three weeks. Based on the daily regressed bodyweights, the participants experienced a loss of 2.5 ± 0.5% of their initial bodyweight. In addition to weight loss, IF has been associated with reduced fasting insulin levels in healthy, non-obese individuals with no change in fasting blood glucose levels [7]. On the other hand, fasting is associated with elevations in free-fatty acids (FFAs) and ketone bodies, which may have opposing effects on inflammation [8].

In a different study, it was also investigated whether IF can be used as a preventive intervention against cardiovascular disease risk factors (CVD) [9] or type 2 diabetes [10]; however, a recent analysis showed that there was no beneficial effect on CVD mortality [11].

Recently, studies have indicated that IF can improve diet adherence in obese participants [12]. Furthermore, IF is a viable alternative to prolonged calorie restriction and offers comparable benefits in terms of weight management and chronic disease management in overweight and obese individuals [13].

Despite numerous studies investigating the positive effects of fasting, there is no study comparing a parallel group design of 16/8 fasting, 20/4 fasting, and ADF in a healthy population or individuals with indications. Using data from a preliminary intervention study, the authors found that even healthy participants found it challenging to adhere to the daily restriction, as social events often led to deviations from the protocol [14].

Therefore, the present study aimed to investigate the feasibility and efficacy of 16/8 fasting, 20/4 fasting, and ADF on metabolic health over an eight-week intervention phase with a controlled-run in phase (four weeks) in a group of healthy individuals.

## 2. Materials and Methods

This EDIF study was conceptualised as a randomised trial with a controlled-run in phase followed by the three fasting interventional groups. The primary outcome of the trial was the change in the BMI. In addition, body composition, metabolic parameters, and blood markers were analysed among and within the respective fasting group. The local ethics committee of the University of Bayreuth (Germany) approved the study protocol (22-014—GB 20 May 2022), which was registered at the German Clinical Trials Register (DRKS00029003).

The study was conducted in conformity with the Declaration of Helsinki and Good Clinical Practice [15]. The potential participants were informed about the study protocol and provided their written informed consent to participate in this trial before any trial-related examinations were performed. This study was designed as a proof-of-concept study for a larger trial.

### 2.1. Eligibility Criteria

Healthy individuals aged 18–65 years with a BMI ≥ 20 kg/m^2^ and a body mass-specific oxygen uptake (VO_2max_) > 20 mL/min/kg were eligible for this study. All potential participants had to be metabolically healthy without any indications of cardiac abnormalities, as assessed by an oral glucose tolerance test (OGTT) and overnight-fasted normal glycaemia (99 mg/dL or lower) [16].

Individuals were excluded if they were simultaneously enrolled in another study or required investigational medicinal products. The blood pressure range of the participants had to be in the range of 90–150 mmHg for systole and 50–95 mmHg for diastole after resting for five minutes in the supine position. Further exclusion criteria included a history of multiple and severe allergies to trial-related products. In addition, significant electrocardiography (ECG) abnormalities, significant bradycardia trend < 35 beats per minute (bpm) at the screening visit, as well as chronic diseases compromising the participant’s safety or compliance with the protocol led to exclusion. The lower limit of <50 bpm heart rate was due to the possibility of including athletes and healthy individuals who exercise regularly, which was mentioned as an inclusion criterion in previous studies [17,18]. In addition, participants with a low heart rate were examined via ECG by a cardiologist to exclude cardiac arrhythmia or conduction disorders. The inclusion and exclusion criteria were assessed during an examination at the screening visit.

### 2.2. Study Design

After a 4-week controlled-run in phase, the participants were randomly assigned to one of the three intervention groups (1:1:1; Randomizer 4.0, Social Psychology Network, Lancaster, PA, USA) [19].

**Figure 1:**
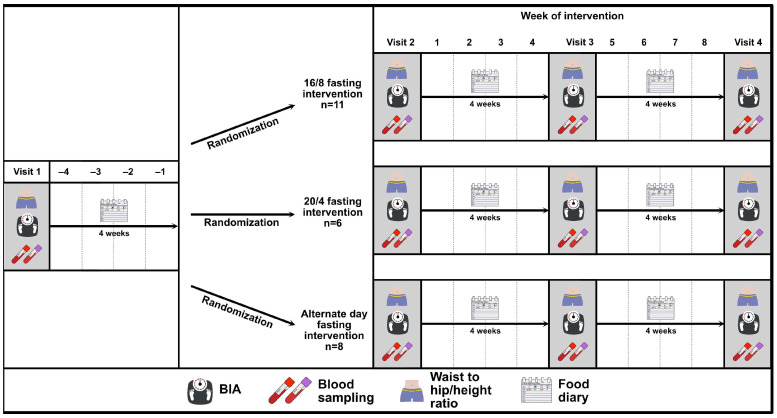
Study flow chart of the EDIF trial, including a controlled-run in phase of four weeks (visit 1 to visit 2) and an interventional phase follow-up of eight weeks (visit 2 to visit 3) after randomisation to the three IF intervention groups: 16/8 fasting, 20/4 fasting, and ADF. Abbreviations: BIA = bioelectrical impedance analysis.

First, a controlled-run in phase was performed by all participants, which was preceded by a baseline visit (visit 1). Visit 2 defined the end of the controlled-run in phase and the beginning of the intermittent fasting phase and visit 3 defined the end of the intermittent fasting phase. The study flow chart is depicted in Figure 1. During each study-related visit, the participants were asked to arrive at the research facility after having fasted overnight, and visits were rescheduled in the case of acute illness.

### 2.3. Analytical Procedures

#### 2.3.1. Anthropometry and Body Composition

A physical examination was performed at each visit, consisting of assessing the general health status via a physical examination, including blood pressure measurement. The body composition (e.g., skeletal muscle mass, fat mass, and fat-free mass) was determined via a bioelectrical impedance analysis (BIA, Inbody 720, Inbody Co., Seoul, Republic of Korea).

In addition, the same investigators determined the waist-to-hip ratio (WHR) twice per visit according to a standardised procedure. The waist circumference was measured with an inelastic 150 cm tape midway between the inferior border of the costal arch and the iliac crest at the midaxillary line. The hip circumference was measured at the maximum posterior projection of the gluteal muscles.

#### 2.3.2. Venous Blood Sampling and Storage

Venous blood was drawn from the median cubital vein. Laboratory analyses comprised a complete blood count and blood cholesterol. Over the active study period, approx. 85 mL of blood was taken and stored pseudonymised at −80 °C after centrifugation at the University of Bayreuth (Department of Exercise Physiology & Metabolism). Blood samples collected were analysed for basic metabolic parameters, including c-reactive protein (CRP), total cholesterol, low-density lipoprotein (LDL), high-density lipoprotein (HDL), triglycerides, ketone bodies, and further various blood markers (e.g., leukocytes, erythrocytes, haemoglobin, haematocrit, mean cell volume (MCV), mean corpuscular haemoglobin (MCH), mean corpuscular haemoglobin concentration (MCHC), red cell distribution width (RDW), mean platelet volume (MPV), thrombocytes, granulocytes, monocytes, and lymphocytes), which were analysed in collaboration with the Clinical Institute for Medical and Chemical Laboratory Diagnostics at the Medical University of Graz.

Concentrations of serum CRP, total cholesterol, HDL, and triglycerides were determined using a cobas 8000 analyzer (Roche Diagnostics GmbH, Mannheim, Germany) with reagents from the same manufacturer. These tests are all traceable to international reference standards. The analytical quality was controlled within daily laboratory routine, and the coefficients of variation for analytical precision were well below the manufacturer’s references. All tests were conducted as instructed by the manufacturer. The concentration of LDL was calculated using the Friedewald equation [20].

The concentrations of ketone bodies in serum (e.g., beta-hydroxy-butyrate) were determined and quantified using a capillary blood sample (mmol/L) from the earlobe in the fasting state (>12 h) with a β-ketone test strip. The test strips were then analysed using the appropriate reader (FreeStyle Libre, Abbott GmbH Abbott Diabetes Care, Wiesbaden, Germany).

#### 2.3.3. Glucose Assessment

- Oral Glucose Tolerance Test (OGTT)

An oral glucose tolerance test (OGTT) was conducted once at the start of each visit to verify an individual’s normal glucose metabolism. The OGTT procedure was followed according to the protocols used in previous studies [21]. The participants received a 300 mL liquid drink containing 75 g glucose (Germania Pharmazeutika GesmbH, Glucoral 75 citron, Wien, Austria). After oral glucose exposition, capillary blood samples were drawn from the earlobe at 0, 15, 30, 60, 90, and 120 min, and glucose was analysed using a point-of-care device (Biosen S-Line, EKF-Diagnostic, Barleben, Germany).

- Continuous Glucose Monitoring (CGM)

Participants received a continuous glucose monitoring system (CGM) to record their glucose levels during the last ten days in the controlled run-in phase and the last ten days during the intervention phase (Dexcom G6, Inc., San Diego, CA, USA). The procedure for using the CGM sensor adhered to protocols established in previous studies [22]. Interstitial glucose levels during the fasting period were assessed using internationally recommended clinical metrics [23]. In addition to the respective glucose ranges, the mean glucose, glucose standard deviation (SD_Gluc_), and coefficient of variation (CV%) were also analysed.

#### 2.3.4. Nutritional Tracking

After visit 1 and visit 2, the participants were provided a food diary and introduced to the food tracker app Fddb (Version 5.0.1 (Build 1)-gms-release, Food Database GmbH, Bremen, Germany). They were asked to record their caloric intake and eating patterns using the app for four consecutive weeks. The participants used the app to record their food intake by scanning barcodes or searching the database, recording the quantities consumed, and the times of consumption. The app created a daily nutritional record in which the food consumed, and times were recorded with a detailed nutritional analysis [24]. The researchers monitored caloric intake via the participants’ accounts and tracked quantity, composition, and timing. The consumed food was also recorded with photographic pictures matched to the app data. We measured adherence using the following procedure: the day was defined as “not fasted” as soon as the participants did not adhere to their fasting days or periods, e.g., for the duration of the fasting period or if they varied their fasting times daily. The participants were instructed to weigh all foods where possible and to record details such as brand names and mealtimes. If weighing was not possible, the food was recorded in household measurements. Emphasis was placed on documenting meals prior to consumption to allow accurate time recording [24]. The participants were able to contact the researcher if they had any questions.

### 2.4. Definition of the Intermittent Fasting Protocols 

#### 2.4.1. The 16/8 Intermittent Fasting (16/8) Protocol

During the 16/8 interval fasting periods, the participants fasted for 16 h; during that time, only water consumption was allowed. Diet drinks, unsweetened tea, and coffee were not allowed. During the remaining eight hours, the participants were allowed to consume any beverage or food of their choice. It was recommended that at least 50% of the fasting time was overnight. There was no fixed requirement to observe the set fasting times. Regarding caloric intake, it was only recommended that the same time slots be chosen each day.

#### 2.4.2. The 20/4 Intermittent Fasting (20/4) Protocol

During the 20/4 interval fasting periods, the participants abstained from caloric intake for 20 h without interruption. Only water consumption was permitted during this period; diet drinks, unsweetened tea, and coffee were prohibited. In the remaining four hours, the participants had the opportunity to have any beverage or food of their choice. It was advised that almost 50% of the fasting duration occurred overnight. Regarding caloric intake, the recommendation was to choose the same timeframe each day, with no fixed requirement for adhering to specific time frames.

#### 2.4.3. Alternate Day Fasting (ADF) Protocol

In the fasting periods occurring on alternate days, the participants abstained from food and beverages for 24 h, and only the consumption of water was allowed. Diet drinks, unsweetened tea, and coffee were not permitted during this fasting period. On the second day, the participants were allowed to consume any beverage or food of their choice.

### 2.5. Statistical Analysis

The data were collected and compiled into Excel (Microsoft Excel Version 16.83 (24031120), Microsoft Corporation, One Microsoft Way, Redmond, WA, USA). Once the data collection was completed, the data were analysed in GraphPad Prism 9.4.1 (GraphPad Software, Inc., San Diego, CA, USA). The data were tested for normal distribution using the Shapiro–Wilk test. When presenting the study results, data are presented according to their distribution as the mean ± standard deviation (SD) or median (interquartile range (IQR) and minima and maxima for the participants’ anthropometric data, metabolic characteristics, and specific glucose data. A two-way analysis of variance (ANOVA) for repeated measures was used to detect interaction differences among participants, considering factors such as group effects. Furthermore, the OGTT data analyses assessed the area under the curve (AUC; trapezoidal rule) using a two-way ANOVA for repeated measurements among the three fasting groups. Subsequently, adjusted post hoc tests using a paired t-test were determined to analyse differences among the respective group means. For baseline characteristics and adherence, the data were analysed using a one-way ANOVA for the beginning and end of the fasting period in adherence. Statistical significance was accepted at *p* < 0.05.

Furthermore, the duration of the intervention was established at eight weeks based on findings from the systematic review and meta-analysis conducted by Cho et al. [25]. This review highlighted studies where the effects of IF remained evident after eight weeks of intervention. Opting for a duration shorter than eight weeks may not have yielded meaningful results, and no significant differences were found with longer intervention periods.

As the EDIF trial operated on a per protocol (PP) principle [26], the participants who deviated from the prescribed protocol were excluded from the final statistical analysis.

## 3. Results

A total of 25 participants of the initially screened 32 potential participants completed the study, with the three visits consisting of the baseline, the controlled-run in phase, and the fasting period. Eleven participants were assigned to the 16/8 (two women) and ADF (four women) groups, and ten participants (five women) were initially assigned to the 20/4 fasting group.

**Figure 2:**
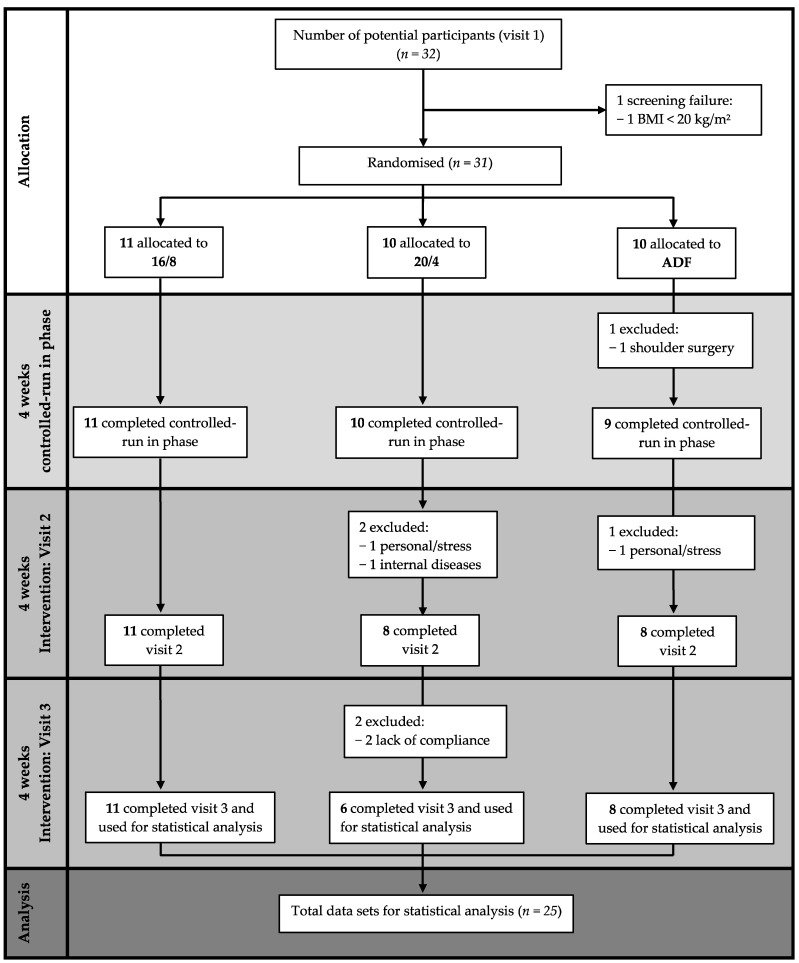
Flow chart considering study-specific features. Abbreviations: 16/8 = fasting for 16 h and eating for the remaining 8 h as time-restricted feeding periods; 20/4 = fasting for 20 h and eating for the remaining 4 h as time-restricted feeding periods; ADF = alternate-day fasting; BMI = body mass index.

The data of *n* = 25 healthy adults (nine women), *n* = 11 in the 16/8 fasting group (two women), *n* = 6 in the 20/4 group (five women), and *n* = 8 in the ADF group (four women) with a mean ± SD age of 25.9 ± 3.1 years and a BMI of 24.8 ± 3.4 kg/m^2^, who completed the EDIF trial, were statistically analysed (Figure 2). The baseline anthropometric data of the participants are presented in Table 1.

### 3.1. Anthropometry

#### 3.1.1. Body Mass Index

**Figure 3:**
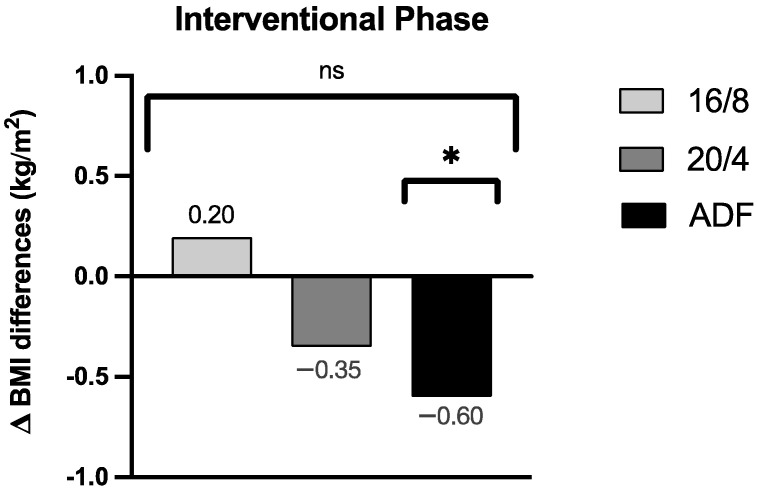
Δ BMI differences of the respective groups during the fasting periods. ns: not significant (*p* > 0.05). *p*-value: * statistically significant (*p* < 0.05). Abbreviation: BMI = body mass index.

By focussing on the data analyses of the BMI, no significant group effect for the BMI assessment could be revealed across the three participating IF cohorts (*p* = 0.30; the results are displayed in Figure 3 as group effect Δ BMI differences). Upon detailed consideration, a pronounced Δ BMI reduction for the ADF participants was obtained during the intervention period (Δ −0.60 ± 0.52 kg/m^2^, *p* = 0.01), but no relevant Δ BMI reductions for the 16/8 (Δ 0.20 ± 0.71 kg/m^2^, *p* = 0.38) and 20/4 (Δ −0.35 ± 0.69 kg/m^2^, *p* = 0.27) cohorts were found. These individually pronounced differences among the three different fasting cohorts are displayed in Figure 3.

#### 3.1.2. Bodyweight

Over the course of the fasting intervention, there were no significant differences in bodyweight among the IF groups (*p* = 0.32). In the 16/8 group, the bodyweight remained unchanged (Δ 0.60 ± 2.2 kg, *p* = 0.39), but the 20/4 IF cohort showed a statistically insignificant weight loss (Δ −1.1 ± 2.1 kg, *p* = 0.26). However, we observed a significant decrease in bodyweight in the ADF cohort (Δ −1.90 ± 1.6 kg, *p* = 0.01).

#### 3.1.3. Waist-to-Hip-Ratio

When analysing the waist-to-hip-ratio (WHR) during the overall course of the study, no significant group effect was observed among all three fasting groups (*p* = 0.32). In the interventional phase, the participants in the ADF cohort (Δ −0.05 ± 0.07, *p* = 0.10) showed no significant changes in the WHR. However, the participants in the 16/8 fasting schedule showed a significant decrease in Δ WHR (Δ −0.04 ± 0.05, *p* = 0.04), as did those in the 20/4 fasting protocol (Δ −0.09 ± 0.05, *p* = 0.05).

### 3.2. Body Composition

#### 3.2.1. Body Fat Mass and Body Fat in Percentage

All participants, regardless of the three intervention groups, showed no significant group effect regarding body fat mass (BFM; *p* = 0.15). The body fat mass in the 16/8 (Δ 0.19 ± 1.86 kg, *p* = 0.74), in 20/4 (Δ −0.37 ± 1.13 kg, *p* = 0.46), and ADF (Δ −0.55 ± 1.16 kg, *p* = 0.22) cohorts did not differ.

Regarding the group effect, the body fat in percentage (BF) values differed but not significantly among the groups (*p* = 0.07). Furthermore, there were no significant differences in the 20/4 fasting protocol (Δ −0.31 ± 1.16%, *p* = 0.54), the 16/8 cohort (Δ 0.01 ± 1.90%, *p* = 0.98), and the ADF cohort (Δ −0.02 ± 1.18% *p* = 0.95) during the interventional phase.

#### 3.2.2. Fat-Free Mass

Analysing the fat-free mass (FFM), the participants showed no significant differences in the group effect (*p* = 0.26). Furthermore, we did not observe any significant differences in FFM in the 16/8 IF cohort (Δ 0.41 ± 1.80 kg, *p* = 0.46) or the 20/4 cohort (Δ −0.74 ± 1.06 kg, *p* = 0.15). Nevertheless, we did observe a significant decrease in the ADF cohort (Δ −1.36 ± 1.48 kg, *p* = 0.04).

#### 3.2.3. Skeletal Muscle Mass

Based on the assessment of the analysed skeletal muscle mass (SMM), no group effect was found across the three intervention groups (*p* = 0.25). The SMM data were comparable in the 16/8 cohort (Δ 0.31 ± 1.04 kg, *p* = 0.36) and the 20/4 group (Δ −0.52 ± 0.61 kg, *p* = 0.09). In contrast, a significant SMM reduction was detected in the ADF group (Δ −0.85 ± 0.91 kg, *p* = 0.03).

### 3.3. Adherence and Food Diary

The participants in the 16/8 fasting group adhered to the fasting intervention 84.5 ± 23.0% of the time. Those in the 20/4 cohort adhered to their self-selected eating window 92.7 ± 9.5% of the time, while those in the ADF cohort adhered approximately 78.1 ± 33.5% of the time; however, no significant differences in adherence were found among the fasting groups (*p* = 0.57).

No statistical group effect was found in caloric intake between the fasting groups throughout the study (*p* = 0.56). At the end of the intervention, in the 16/8 cohort, the caloric intakes were nearly similar (Δ −119.2 ± 444.6 kcal, *p* = 0.42). Furthermore, there were no differences in the 20/4 protocol (Δ −36.17 ± 293.3 kcal, *p* = 0.77). In contrast, the ADF cohort (Δ −481.6 ± 270.1 kcal, *p* = 0.002) showed a significant decrease.

When analysing the macronutrient intake, no significant group effects were observed during the intervention phase (the results are displayed in Table 2). Some significant differences in the initial macronutrients in the respective fasting group are shown in Table 2.

### 3.4. Metabolic and Inflammatory Markers

#### 3.4.1. C-Reactive Protein (CRP)

Throughout the fasting intervention, there was no group effect (*p* = 0.25) in CRP levels among all fasting groups. Upon detailed consideration, no significant differences were found among the fasting groups: 16/8 IF cohort (Δ −3.25 ± 11.87 mg/L, *p* = 0.41), 20/4 cohort (Δ −2.20 ± 2.56 mg/L, *p* = 0.19), and the ADF cohort (Δ 1.10 ± 23.03 mg/L, *p* = 0.92).

#### 3.4.2. Blood Cholesterol

When analysing the total cholesterol over the course of the study, no statistically significant group effects were observed among the three study groups (*p* = 0.98). In each group, a significant increase in the total cholesterol was observed by the participants in the 16/8 cohort (Δ 15.10 ± 13.45 mg/L, *p* = 0.006). The 20/4 IF cohort (Δ 16.75 ± 11.53 mg/L, *p* = 0.06) and the ADF cohort (Δ 22.80 ± 23.48 mg/L, *p* = 0.10) both had no significant differences in the total cholesterol.

Regarding high-density lipoprotein (HDL), all study groups had no significant differences (*p* = 0.33). During the intervention phase, the HDL values in the 16/8 (Δ 3.60 ± 7.03 mg/L, *p* = 0.14), 20/4 (Δ 10.75 ± 9.03 mg/L, *p* = 0.10), and in ADF (Δ 2.40 ± 7.50 mg/L, *p* = 0.51) cohorts did not differ.

By focussing on the data analyses for low-density lipoprotein (LDL), no significant group effect for the LDL assessment was observed across the three participating cohorts (*p* = 0.30). However, the LDL levels increased significantly during the intervention phase in the 16/8 (Δ 11.00 ± 10.97 mg/L, *p* = 0.01) and of 20/4 (Δ 11.00 ± 3.37 mg/L, *p* = 0.01) IF cohorts. In contrast, the ADF cohort (Δ 25.40 ± 21.45 mg/L, *p* = 0.06) remained unaffected.

#### 3.4.3. Triglycerides

Based on the analysed triglycerides, no significant group effect was found among the fasting groups (*p* = 0.45). Furthermore, all intervention groups showed no significant differences in triglycerides during the respective fasting phase: 20/4 cohort (Δ −24.00 ± 23.19 mg/dL, *p* = 0.13), ADF (Δ −22.80 ± 40.31 mg/dL, *p* = 0.27), and in the 16/8 cohort (Δ 3.80 ± 43.20 mg/dL, *p* = 0.79).

#### 3.4.4. Ketone Bodies

All analysed participants showed no group effect in the ketone body assessment (*p* = 0.21). The 16/8 IF cohort (Δ −0.04 ± 0.08 mmol/L, *p* = 0.17) and the 20/4 cohort (Δ −0.03 ± 0.05 mmol/L, *p* = 0.17) were nearly constant in ketones. In addition, there were no differences in the ADF cohort (Δ −0.01 ± 0.16 mmol/L, *p* = 0.84).

#### 3.4.5. Blood Count

In the appendices, Table A1 shows no significant group effect (*p* > 0.05) for other blood count parameters over the course of the study. This included leukocytes, erythrocytes, haemoglobin, haematocrit, MCV, MCH, MCHC, RDW, MPV, thrombocytes, granulocytes, monocytes, and lymphocytes. Upon detailed consideration, significant Δ differences can be revealed within the three participating fasting groups during each interventional phase (*p*-value *, statistically significant: *p* < 0.05).

### 3.5. Glycaemia

#### 3.5.1. CGM-Derived Glycaemia

Based on the mean interstitial glucose levels obtained via CGM (Dexcom G6), no group effects were found among all three fasting groups (*p* = 0.80). Mean interstitial glucose levels were similar in comparison to the three fasting periods. In the 16/8 cohort (Δ −0.20 ± 12.79 mg/dL, *p* = 0.97), the mean interstitial glucose levels remained stable. Furthermore, the mean interstitial glucose levels did not differ significantly in the 20/4 cohort (Δ −2.50 ± 14.85 mg/dL, *p* = 0.85) and in the ADF cohort (Δ −4.20 ± 11.48 mg/dL, *p* = 0.46).

For the SD_Gluc_ of interstitial glucose, no significant differences were observed among all study groups (*p* = 0.44). Analysing the SD_Gluc_ of interstitial glucose in each study cohort, no significant differences were seen: 16/8 (Δ 1.40 ± 7.02 mg/dL, *p* = 0.68); 20/4 (Δ −2.50 ± 2.12 mg/dL, *p* = 0.34); and ADF (Δ 3.20 ± 4.03 mg/dL, *p* = 0.15).

The coefficient of variation (CV%) of interstitial glucose showed no significant alteration among all study cohorts (*p* = 0.31). The evaluation of the interstitial glucose values by the CV% within the respective fasting group showed no significant differences (16/8, Δ 1.06 ± 4.37%, *p* = 0.62; 20/4, Δ 3.70 ± 7.07%, *p* = 0.59; and ADF, Δ 1.72 ± 4.02% *p* = 0.39).

Other interstitial glucose parameters, as shown in Table 3, remained similar among all four trial arms (*p* > 0.05).

#### 3.5.2. Glycaemia during OGTT

Throughout the OGTT, no significant group effect in the plasma glucose levels was found across all three participating fasting groups (*p* = 0.40).

The median [interquartile range] values of the 120-minute OGTT plasma glucose AUC were similar when comparing all intervention regimes (16/8, total area Δ −452 [−560–(−344)], *p* = 0.72; 20/4, total area Δ 856 [1027–745], *p* = 0.08; and ADF, total area Δ 635 [−301–1570], *p* = 0.33).

Over the course of the OGTT, the mean plasma glucose levels showed no significant group effect across the fasting groups (*p* = 0.12). Furthermore, the mean 120-minute plasma glucose levels did not differ significantly in all fasting cohorts: 16/8 (Δ −1.39 ± 8.89 mg/dL, *p* = 0.71); 20/4 (Δ 4.76 ± 4.90 mg/dL, *p* = 0.06); and ADF (Δ 5.20 ± 7.13 mg/dL, *p* = 0.13).

## 4. Discussion

To the best of our knowledge, EDIF is the first study comparing the efficacy and feasibility of different IF interventions (16/8, 20/4, and ADF) using anthropometric parameters, body composition assessment, as well as metabolic and blood measurements in healthy individuals during eight weeks of various fasting protocols with a controlled-run in phase.

Our results suggest that different fasting intervention protocols are not associated with a group effect on the BMI. The results of the EDIF trial partly differ from those reported by Varady [13], where an ADF fasting method or daily calorie restriction (between 15% and 60% of the usual daily calorie intake) was conducted. In this case, the overall weight loss in overweight or obese individuals was similar between the groups of each dietary pattern. This tendency is supported by a study comparing continuous and intermittent energy restriction over four weeks in obese men [5]. In this case, it was found that a more significant weight loss was achieved with intermittent energy restriction compared to continuous energy restriction. Moreover, these trends of short-term IF on weight loss in individuals have been evaluated in further various small clinical trials. In one of these studies conducted by Cai et al., participants with non-alcoholic fatty liver followed a 12-week ADF or 16/8 fasting program, with ad libitum caloric intake during the feasting phase, which resulted in a significant weight loss in both fasting programs compared to the control group [27]. These trends in bodyweight reduction were confirmed by a review by Cienfuegos et al., who summarised that TRF leads to modest weight loss (1 to 4% from baseline) in people with type 2 diabetes when caloric intake is restricted to 4–10 h/day [28]. However, in our EDIF trial, we only achieved a significant reduction in the BMI (*p* = 0.01) and bodyweight (*p* = 0.01) within the ADF cohort in a healthy population. In this context, the reduction in the BMI during the intervention phase in the ADF group can be explained by the significantly reduced intake of calories and the individual macronutrients.

Previous research on the impact of IF and other body composition parameters revealed contrary findings. In some studies of obese individuals, 16/8 fasting protocols have been shown to reduce BF [29], while other TRF regimens, such as 16/8 or 12 h fasting protocols, have not [30,31]. Recent trials showed that TRF with a 16/8 fasting protocol led to reduced caloric intake and prevented gains in body lean mass [32]. In three meta-analysis studies [5,33,34], IF was associated with a more significant reduction in fat mass among the participants, while two of those studies indicated a greater loss of lean body mass in the IF intervention group [34,35]. In contrast, the data of our EDIF trial showed no significant differences across the three participating fasting groups in BFM values (*p* = 0.15) over the eight-week fasting period. Our study indicated that BFM remained almost constant in the 16/8 (*p* = 0.74), 20/4 (*p* = 0.46), and ADF (*p* = 0.22) cohorts and did not differ during the interventional phase. Furthermore, no group effect was observed for SMM between the study groups (*p* = 0.25). There appeared to be an insignificant trend towards an increase in SMM in both the 16/8 (*p* = 0.36) and 20/4 (*p* = 0.09) cohorts. The ADF cohort, on the other hand, was the only fasting group to show a significant decrease in SMM (*p* = 0.03) and FFM (*p* = 0.04).

Concerning waist circumference, the systematic review conducted by Seimon et al. [36] reported comparable reductions in both intervention groups (calorie energy restriction and IF). Moreover, the study by Arguin et al. [33] revealed a greater reduction in waist circumference in the intermittent diet and continuous diet group. In our results, no significant group effect in the WHR was observed throughout the trial (*p* = 0.32). Therefore, based on the observed results, it can be concluded that WHR changes in the short term (<8 weeks) do not differ among IF regimens. Upon closer examination at the WHR, the participants in the ADF cohort showed no significant differences during the fasting period (*p* = 0.10), while significant decreases in the 16/8 cohort (*p* = 0.04) and in the 20/4 cohort (*p* = 0.05) were observed.

Regarding the feasibility of the three interventions, there was no significant group effect in adherence across the specific fasting interventions (*p* = 0.57). Despite the importance of lifestyle interventions to improve health, in practice, adherence is often limited [37]. Indeed, concerning the feasibility of the three interventions, the self-reported adherence to the specific interventions varied among the groups. The 20/4 cohort had the highest adherence (93%), whereas the 16/8 (85%) and the ADF groups (78%) had lower adherences, suggesting that adherence is difficult even among healthy people. Further studies need to focus on populations with specific health conditions to shed light on whether the adherence observed among healthy people persists or worsens in individuals who are already burdened by a disease. For the practical application of fasting in health care, it is crucial to address the problem of low adherence. Hence, strategies to improve adherence must be investigated and tailored to different populations to meet their specific needs. In our EDIF trial, a possible deficiency in adherence was revealed by the self-assessment of foods in the Fddb app. Therefore, food could have been entered incorrectly, which could have led to missing caloric intake or macronutrient distribution.

In addition, our findings did not show that IF provides additional benefits regarding metabolic or blood level variables compared to fasting protocols, which is consistent with the findings of previous clinical trials [38,39]. Upon closer examination at the haematological markers, the values of the total cholesterol showed a significant increase in the participants of the 16/8 cohort (*p* = 0.006). Furthermore, there were no relevant indications of significant differences in HDL levels. The group effect in HDL across the study groups in the EDIF trial was not statistically significant (*p* = 0.33). However, conducting a more comprehensive study could shed additional clarity on whether the observed trend towards an increase in HDL levels was coincidental. It should be noted that a significant increase in LDL levels was observed in both the 16/8 cohort (*p* = 0.01) and the 20/4 cohort (*p* = 0.01), although this result was considered irrelevant. The prevalence of CVD and metabolic disorders has been increasing globally in recent years, attributed to suboptimal metabolic and hormonal factors, as well as rising stress levels [40]. Our EDIF trial did not show an increased risk of metabolic derailments as determined by the evaluation of the OGTT and the wearing of the CGMs. However, caution is needed when interpreting these observations for several reasons. First, it is known that metabolic changes during fasting and dieting periods may vary from individual to individual. Second, blood glucose levels may be affected by changes in activity due to circadian rhythms. Finally, metabolic changes also play a role in fasting-specific changes in glycaemia [38].

The potential of IF for weight loss and improved metabolic health is consistent with the principles of precision medicine, particularly in overweight or obese individuals. While most clinical studies investigating IF in humans have observed a reduction in certain risk parameters for diseases such as diabetes mellitus, it is important to note that there are exceptions to this trend. For some individuals, IF can lead to significant improvements, while for others, only minimal changes are observed, highlighting the need for personalised precision medicine [41,42]. In addition, some studies suggest that IF can provide a valuable contribution to weight loss programmes. However, it is important to consider the composition of the individual’s diet and calorie density in such fasting interventions [37]. Our preliminary reporting has several limitations. First, the number of participants included was relatively small (*n* = 25); therefore, the data obtained in our study should be considered as hypothesis-generating, and further investigations should focus on a larger sample of participants to verify the scientific evidence base. Second, the study was relatively short (twelve weeks). Longer-term trials will be needed to determine the degree of bodyweight loss that can be achieved with IF. Third, adherence and dietary intake were self-completed and assessed using a mobile phone app; therefore, more data maintenance was often needed. Thus, food intake and calorie deficit estimates need to be more accurate. Fourth, the dropout rate was very high, so that no direct comparison of the individual groups was possible in a meaningful way. Fifth, we could have added another intervention group with a traditional hypocaloric diet in our trial to provide further evidence of the effects of IF compared to the continuous caloric restriction diet; see the results of IF compared to caloric restriction in the study by Zhang et al. [43]. Sixth, we did not analyse the power of our sample size. Due to the characteristics of the pilot study, we initially focussed on a small sample size. In subsequent studies, the data from this EDIF trial will be used to conduct a power analysis. Seventh, our study examined only healthy individuals. Therefore, our results cannot be generalised to other populations. Furthermore, in a study with a larger sample, a differentiation by gender can also be made in the sub-analysis.

## 5. Conclusions

This primary outcome analysis from the EDIF trial offers valuable insights into the potential metabolic health advantages of various IF protocols, such as 16/8, 20/4, or ADF, among healthy individuals.

In summary, IF appears to be efficiently transferable to the daily life of healthy individuals and showed positive trends for metabolic health during our intervention phase with different fasting protocols [44,45]. However, it is noteworthy that strict adherence to fasting protocols poses challenges even for the healthy population.

## Figures and Tables

**Table 1:** Participants´ baseline anthropometric characteristics.

Parameter	Overall (*n* = 25)	16/8 (*n* = 11)	20/4 (*n* = 6)	ADF (*n* = 8)	*p*-Values
Age (y)	25.9 ± 3.1	26.3 ± 4.1	25.8 ± 2.1	25.4 ± 2.1	0.94
Bodyweight (kg)	78.0 ± 14.1	79.4 ± 18.4	72.5 ± 10.4	76.8 ± 9.2	0.77
Height (cm)	177.0 ± 8.5	177.8 ± 11.3	176.0 ± 6.5	176.4 ± 6.4	0.80
BMI (kg/m^2^)	24.8 ± 3.4	25.0 ± 4.5	23.2 ± 2.2	24.7 ± 2.4	0.90
Waist-to-hip-Ratio	0.91 ± 0.01	0.89 ± 0.03	0.88 ± 0.06	0.86 ± 0.03	0.56

Data are presented as the mean ± SD. No significant differences were obtained by one-way ANOVA testing across the three participating fasting cohorts. Abbreviations: 16/8 = fasting for 16 h and eating for the remaining 8 h as time-restricted feeding periods; 20/4 = fasting for 20 h and eating for the remaining 4 h as time-restricted feeding periods; ADF = alternate-day fasting.

**Table 2:** Differences in macronutrient intake during nutritional tracking via the food tracker app Fddb.

Parameter	Cohort	Δ Differences during Interventional Phase	*p*-Value (Respective Cohort)	*p*-Value (Group Effect)
Carbohydrates (kcal)	16/8	Δ −96.85 ± 193.7	0.12	0.41
	20/4	Δ −141.0 ± 156.4	0.04 *	
	ADF	Δ −256.2 ± 249.1	0.04 *	
Proteins (kcal)	16/8	Δ −10.16 ± 58.37	0.60	0.22
	20/4	Δ 25.67 ± 42.27	0.16	
	ADF	Δ −115.7 ± 49.82	0.001 *	
Fats (kcal)	16/8	Δ −60.96 ± 189.2	0.34	0.16
	20/4	Δ 10.11 ± 126.6	0.83	
	ADF	Δ −154.4 ± 105.0	0.008 *	

Data are presented as a the Δ mean ± SD. No significant group effect was obtained by two-way ANOVA testing across the three participating fasting cohorts. Significant Δ differences were observed during the intervention phase in the respective fasting group. *p*-value: * statistically significant (*p* < 0.05). Abbreviations: kcal = kilocalorie; 16/8 = fasting for 16 h and eating for the remaining 8 h as time-restricted feeding periods; 20/4 = fasting for 20 h and eating for the remaining 4 h as time-restricted feeding periods; ADF = alternate-day fasting.

**Table 3:** Comparison of glycaemic ranges for all groups using CGM.

Glycaemic Range	16/8 (*n* = 11)	20/4 (*n* = 6)	ADF (*n* = 8)	*p*-Value (Group Effect)
TAR (%)	0 ± 0	0 ± 0	0 ± 0	0.99
TIR (%)	98 ± 1	98 ± 1	97 ± 3	0.85
TBR (%)	1 ± 0	2 ± 1	2 ± 2	>0.99

Data are presented as percentages. No significant group effect was obtained by two-way ANOVA testing across the three participating fasting cohorts. Abbreviations: 16/8 = fasting for 16 h and eating for the remaining 8 h as time-restricted feeding periods; 20/4 = fasting for 20 h and eating for the remaining 4 h as time-restricted feeding periods; ADF = alternate-day fasting; TAR = time above range (>181 mg/dL; 10.1–13.9 mmol/L); TBR = time below range (<70 mg/dL; 3.0–3.9 mmol/L); TIR = time in range (70–180 mg/dL; 3.9–10.0 mmol/L).

## Data Availability

The data are available upon reasonable request.

## Appendix A

**Table A1:** Differences in Δ haematological markers during the intervention phase due to different fasting interventions.

Parameter	Cohort	Δ Differences during Interventional Phase	*p*-Value (Respective Cohort)	*p*-Value (Group Effect)
Leukocytes (g/L)	16/8	Δ −0.05 ± 1.23	0.90	0.41
	20/4	Δ −0.88 ± 1.60	0.23	
	ADF	Δ −0.70 ± 0.38	0.001 **	
Erythrocytes (g/L)	16/8	Δ −0.05 ± 0.20	0.44	0.28
	20/4	Δ −0.25 ± 0.52	0.30	
	ADF	Δ −0.03 ± 0.17	0.68	
Haemoglobin (g/dL)	16/8	Δ −0.08 ± 0.51	0.64	0.61
	20/4	Δ −0.24 ± 1.50	0.254	
	ADF	Δ −0.06 ± 0.38	0.66	
Haematocrit (%)	16/8	Δ −0.20 ± 1.81	0.74	0.45
	20/4	Δ −2.67 ± 3.98	0.16	
	ADF	Δ −0.50 ± 1.41	0.35	
MCV (fl)	16/8	Δ −0.10 ± 1.37	0.82	0.09
	20/4	Δ 0.50 ± 2.59	0.66	
	ADF	Δ −0.63 ± 2.13	0.43	
MCH (pg)	16/8	Δ −0.10 ± 0.57	0.59	0.29
	20/4	Δ −0.17 ± 0.75	0.61	
	ADF	Δ −0.13 ± 0.64	0.60	
MCHC (g/dL)	16/8	Δ −0.10 ± 0.57	0.59	0.66
	20/4	Δ −0.50 ± 1.05	0.30	
	ADF	Δ 0.38 ± 0.74	0.20	
RDW (%)	16/8	Δ −0.12 ± 0.43	0.40	0.29
	20/4	Δ 0.27 ± 0.73	0.41	
	ADF	Δ −0.28 ± 0.41	0.10	
Thrombocytes (g/L)	16/8	Δ 3.10 ± 22.30	0.67	0.08
	20/4	Δ 1.67 ± 33.00	0.91	
	ADF	Δ −14.13 ± 26.42	0.17	
MPV (fl)	16/8	Δ −0.20 ± 0.42	0.17	0.13
	20/4	Δ −0.17 ± 0.41	0.36	
	ADF	Δ 0.25 ± 0.46	0.17	
Neutrophile granulocytes (g/L)	16/8	Δ −0.02 ± 1.06	0.95	0.25
	20/4	Δ −0.72 ± 1.49	0.29	
	ADF	Δ −0.50 ± 0.45	0.02 *	
Immature granulocytes (g/L)	16/8	Δ −0.001 ± 0.01	0.76	0.30
	20/4	Δ 0.00 ± 0.02	>0.99	
	ADF	Δ −0.01 ± 0.01	0.10	
Eosinophile granulocytes (g/L)	16/8	Δ 0.03 ± 0.07	0.25	0.10
	20/4	Δ −0.04 ± 0.03	0.02 *	
	ADF	Δ −0.04 ± 0.05	0.07	
Basophile granulocytes (g/L)	16/8	Δ 0.00 ± 0.01	>0.99	0.23
	20/4	Δ −0.01 ± 0.01	0.03 *	
	ADF	Δ −0.01 ± 0.01	0.17	
Monocytes (g/L)	16/8	Δ −0.06 ± 0.18	0.35	0.34
	20/4	Δ −0.07 ± 0.07	0.045 *	
	ADF	Δ −0.05 ± 0.12	0.27	
Lymphocytes (g/L)	16/8	Δ 0.01 ± 0.33	0.96	0.11
	20/4	Δ −0.05 ± 0.15	0.41	
	ADF	Δ −0.10 ± 0.47	0.57	

Data are presented as the Δ mean ± SD. By two-way ANOVA testing across the three participating fasting cohorts, no significant group effect was obtained. Significant Δ differences were revealed in each fasting group during the interventional phase. *p*-value: statistically significant at * *p* < 0.05 and ** *p* < 0.01. Abbreviations: MCV = mean corpuscular/cell volume; MCH = mean corpuscular haemoglobin; MCHC = mean corpuscular haemoglobin concentration; RDW = red cell distribution width; MPV = mean platelets volume; 16/8 = fasting for 16 h and eating for the remaining 8 h as time-restricted feeding periods; 20/4 = fasting for 20 h and eating for the remaining 4 h as time-restricted feeding periods; ADF = alternate-day fasting.
